# Optimizing Cardiovascular Risk in PLWH: Real-World Efficacy and Lipid Kinetics of Fixed-Dose Combination Rosuvastatin Ezetimibe

**DOI:** 10.3390/idr18050119

**Published:** 2026-10-16

**Authors:** Katia Falasca, Claudio Ucciferri, Damiano D’Ardes, Raffaele Ferri, Livia Moffa, Francesca Chiappini, Riccardo Mattia Ricciardi, Francesco Cipollone, Jacopo Vecchiet

**Affiliations:** 1 Clinic of Infectious Diseases, Department of Medicine and Aging Science, “G. d’Annunzio” University of Chieti-Pescara, 66100 Chieti, Italy; ferri.raffaele22@gmail.com (R.F.); moffalivia1@gmail.com (L.M.); francesca.chiappini@studenti.unich.it (F.C.); jacopo.vecchiet@unich.it (J.V.); 2 Infectious Disease Clinic, Department of Medicine and Surgery, University of Perugia, 06132 Perugia, Italy; claudio.ucciferri@unipg.it; 3 Institute of Internal Medicine, Department of Medicine and Aging Science, “G. d’Annunzio” University of Chieti-Pescara, 66100 Chieti, Italy; damiano.dardes@unich.it (D.D.); riccardomattiaricciardi@gmail.com (R.M.R.); francesco.cipollone@unich.it (F.C.)

**Keywords:** HIV, dyslipidemia, rosuvastatin, ezetimibe, LDL cholesterol, cardiovascular risk

## Abstract

Background: People living with HIV (PLWH) have increased cardiovascular disease risk, and dyslipidemia remains a major modifiable factor. Achieving recommended low-density lipoprotein cholesterol (LDL-C) targets can be challenging. Objective: To evaluate the real-world effectiveness and safety of fixed-dose low-dose rosuvastatin/ezetimibe in virologically stable PLWH and to describe lipid-lowering kinetics in comparison with an HIV-negative control cohort. Methods: This retrospective single-center study included 32 PLWH and 43 HIV-negative outpatients receiving rosuvastatin 5 mg plus ezetimibe 10 mg daily. Lipid parameters, cardiovascular risk scores, and safety markers were assessed at baseline, 3 months, and 6 months. Results: In PLWH, LDL-C decreased from 170.7 ± 35.1 to 87.8 ± 24.6 mg/dL at 6 months, corresponding to a mean reduction of 46.7% (*p* < 0.001). Total cholesterol and non-HDL cholesterol also decreased significantly (*p* < 0.001 for both), while triglycerides decreased significantly at month 6 (*p* = 0.016). Overall, 53.1% of PLWH reached guideline-recommended LDL-C targets. Both the 5-year and 10-year D:A:D scores decreased significantly during follow-up (*p* < 0.001 for both). The exploratory comparison suggested a more rapid early lipid response among HIV-negative controls, with most of the reduction occurring by month 3. No treatment-limiting adverse events or viro-immunological deterioration occurred. Conclusions: Low-dose rosuvastatin combined with ezetimibe was associated with substantial improvement in atherogenic lipid parameters in virologically stable PLWH, while the exploratory finding of an earlier response among HIV-negative controls may support further investigation of treatment-response kinetics.

## 1. Introduction

The advent of highly effective combination antiretroviral therapy has transformed HIV infection into a manageable chronic condition and increased life expectancy [1,2,3]. Cardiovascular risk in PLWH is multifactorial and includes chronic immune activation, persistent inflammation, metabolic disturbances, traditional risk factors, and the effects of long-term antiretroviral therapy. PLWH frequently present with elevated LDL-C, hypertriglyceridemia, and reduced HDL-C [4]. Dyslipidemia is therefore an important modifiable target in long-term HIV care [5,6,7,8].

Statins remain the cornerstone of lipid management, but their use in PLWH requires careful consideration of antiretroviral regimens and potential drug–drug interactions [9,10]. Current cardiovascular and HIV guidelines emphasize achievement of appropriately intensive LDL-C targets, particularly in patients at high or very high cardiovascular risk [5]. Combination treatment with a statin and ezetimibe may be considered when a substantial LDL-C reduction is required and escalation of the statin dose is undesirable [11,12,13,14,15,16,17].

Combination treatment with a statin and ezetimibe has been evaluated in high-risk dyslipidemia and in selected HIV cohorts. Available evidence suggests that adding ezetimibe to rosuvastatin may provide additional LDL-C lowering in patients who require a substantial reduction, but treatment response is influenced by baseline risk, previous therapy, adherence, and the selected comparator. These data support an individualized treatment strategy and do not establish universal superiority over statin monotherapy [13,18].

The primary aim of this study was to assess the 6-month LDL-C reduction and target attainment associated with fixed-dose rosuvastatin/ezetimibe in virologically stable PLWH. Secondary aims were to evaluate short-term tolerability, cardiovascular risk estimates, and exploratory differences in lipid-lowering trajectories compared with HIV-negative controls.

## 2. Materials and Methods

### 2.1. Study Design and Patients

This retrospective study was conducted among outpatients followed at the Infectious Diseases Clinic of the University “G. d’Annunzio” SS. Annunziata Hospital in Chieti between 1 January 2024 and 31 December 2024. We included adults with HIV-1 infection for at least 60 months, stable and effective antiretroviral therapy, and persistently elevated LDL-C requiring lipid-lowering treatment. Eligibility required LDL-C > 130 mg/dL or LDL-C > 100 mg/dL in the presence of cardiovascular risk factors on at least two separate qualifying measurements. Patients with acute infection, recent corticosteroid or NSAID exposure, pregnancy, recent ischemic events, severe liver-enzyme elevation, or severe creatine-kinase elevation were excluded.

All PLWH initiated rosuvastatin 5 mg plus ezetimibe 10 mg daily de novo. The decision to initiate the combination rather than statin monotherapy was made in routine clinical practice because baseline LDL-C was substantially elevated and a large LDL-C reduction was expected to be necessary to reach the recommended target. The low-dose combination was selected to provide complementary LDL-C lowering without escalating the rosuvastatin dose. This was not a randomized comparison with statin monotherapy.

A control group comprising 43 HIV-negative individuals was included for exploratory comparative analysis. Controls were consecutively enrolled from the metabolic diseases outpatient clinic of the same institution during the same period. They were not individually matched to PLWH for age or sex. All HIV-negative controls were naïve to lipid-lowering treatment and initiated rosuvastatin/ezetimibe de novo. They were selected because they had a comparable clinical indication for initiating the same regimen and a broadly comparable overall cardiovascular risk burden requiring lipid-lowering therapy.

This approach was considered clinically relevant because HIV infection is itself a cardiovascular risk modifier. Persistent immune activation, chronic inflammation, antiretroviral exposure, and HIV-associated metabolic abnormalities may contribute to cardiovascular risk beyond conventional demographic and metabolic factors. Baseline Framingham 10-year risk was comparable between PLWH and controls (19.6 ± 18.7% versus 20.4 ± 14.3%, respectively). Because the cohorts differed in age, sex, BMI, and some comorbidities, the between-group analysis was considered exploratory and residual confounding could not be excluded.

The first clinical visit did not necessarily coincide with treatment initiation. Treatment was initiated after repeated qualifying LDL-C measurements and cardiovascular risk assessment. T0 was defined as the laboratory assessment immediately preceding or closest to treatment initiation after eligibility had been confirmed. The definitive analytical workbook did not contain the exact dates required to calculate a fixed interval between the qualifying measurements and treatment initiation; this limitation is acknowledged and no unverified interval is reported.

### 2.2. Plasma Samples

After overnight fasting, the following biomarkers were measured: complete blood count, creatinine, blood urea nitrogen, estimated glomerular filtration rate, AST, ALT, gamma-glutamyl transferase, alkaline phosphatase, lactate dehydrogenase, total cholesterol, LDL-C, HDL-C, triglycerides, cystatin C, sodium, potassium, uric acid, creatine kinase, and 25-hydroxyvitamin D. CD4+ and CD8+ T-cell counts were determined by flow cytometry using a FACSCanto II flow cytometer (BD Bio-sciences, San Jose, CA, USA). Plasma HIV-1 RNA levels were determined using the AMPLICOR HIV-1 MONITOR assay (Roche Molecular Sys-tems, Inc., Pleasanton, CA, USA).

At baseline, 53.1% of PLWH received an INSTI-based regimen combined with an NRTI backbone. The remaining 46.9% of PLWH received an unboosted dual-therapy regimen. No participant received a ritonavir- or cobicistat-boosted protease-inhibitor regimen. Because of the limited sample size, regimen-specific stratification and covariate-adjusted ART analyses were not performed.

Cardiovascular risk estimates were calculated using the latest available full D:A:D cardiovascular risk model (2016 update), which provides 5-year and 10-year estimates and incorporates total cholesterol, HDL-C, demographic, clinical, immunological, and antiretroviral-related variables. LDL-C is not entered as a separate direct variable in the D:A:D equation.

Ethical approval, data protection, and consent procedures are described in the dedicated ethics statement. Data were pseudonymized and analyzed in accordance with the Declaration of Helsinki and applicable institutional requirements.

### 2.3. Statistical Analysis

The primary endpoint was the percentage change in LDL-C from baseline to month 6 among PLWH. Secondary endpoints included absolute LDL-C change, LDL-C target attainment, changes in total cholesterol, non-HDL cholesterol, HDL-C, triglycerides, cardiovascular-risk estimates, and safety parameters. Quantitative variables are summarized as mean ± standard deviation and qualitative variables as frequency and percentage. Within-PLWH changes over time were evaluated using repeated measures analyses. For D:A:D scores, overall changes across T0, T3, and T6 were assessed using the Friedman test, followed by Wilcoxon signed-rank tests for pairwise comparisons. The longitudinal response between PLWH and HIV-negative controls was explored using mixed-design analysis of variance. Risk-score correlations were assessed using Spearman’s coefficient. Because of the retrospective design and small sample size, the between-group and correlation analyses were considered exploratory, and nominal *p*-values were interpreted without causal inference. No adjustment for multiplicity was prespecified. Given the limited sample size, a multivariable longitudinal model simultaneously adjusting for age, sex, BMI, diabetes, hypertension, baseline LDL-C, baseline cardiovascular risk, and ART regimen was not considered statistically stable. The between-group analysis was therefore retained as an unadjusted exploratory analysis to avoid overfitting and imprecise estimates. Statistical analyses were performed using IBM SPSS Statistics for Windows, version 29.0 (IBM Corp., Armonk, NY, USA).

## 3. Results

A total of 32 PLWH and 43 HIV-negative controls were included. The PLWH cohort included 23 males (71.9%), with a mean age of 54.0 ± 7.4 years and mean BMI of 25.9 ± 5.3 kg/m^2^. Controls were older and included a lower proportion of males. All controls were naïve to previous lipid-lowering treatment and initiated rosuvastatin/ezetimibe de novo. Baseline Framingham risk was comparable between groups, although demographic and metabolic differences remained and the comparison was therefore exploratory (Table 1).

In PLWH, total cholesterol decreased from 236.4 ± 39.2 mg/dL at baseline to 175.2 ± 38.1 mg/dL at month 3 and 153.3 ± 30.1 mg/dL at month 6 (*p* < 0.001). LDL-C decreased from 170.7 ± 35.1 mg/dL to 111.0 ± 41.2 mg/dL and 87.8 ± 24.6 mg/dL, respectively (*p* < 0.001). Non-HDL cholesterol decreased from 187.4 ± 38.4 mg/dL to 127.7 ± 35.7 mg/dL at month 3 and 104.7 ± 25.8 mg/dL at month 6 (*p* < 0.001). Triglycerides decreased from 167.1 ± 127.4 mg/dL to 113.4 ± 82.1 mg/dL at month 6 (*p* = 0.016), while HDL-C remained stable. Overall, 53.1% reached the LDL-C target recommended for their cardiovascular-risk category.

**Figure 1:**
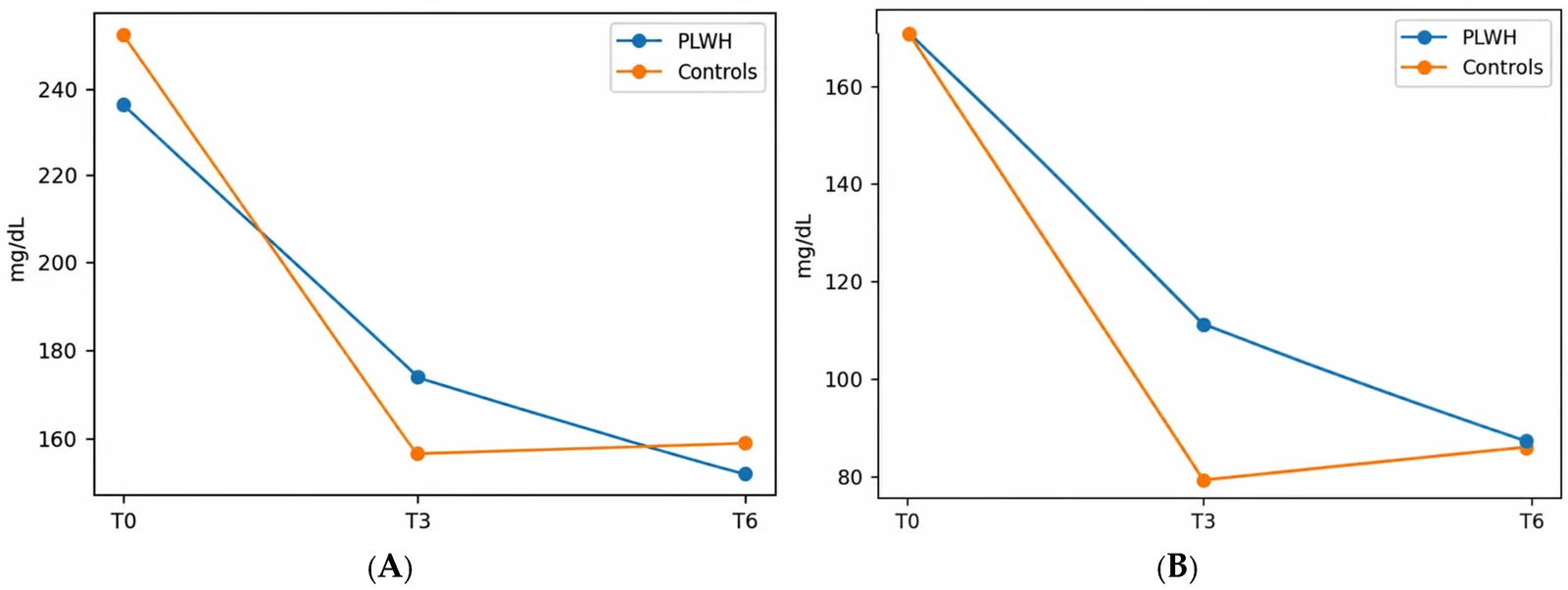
Longitudinal evolution of total cholesterol (**A**) and LDL cholesterol (**B**) in people living with HIV (PLWH) and HIV-negative controls treated with rosuvastatin/ezetimibe. The exploratory comparison suggested a more rapid early lipid response among HIV-negative controls, with most of the reduction occurring within the first three months and subsequent stabilization, whereas PLWH showed a more gradual but sustained decline over the six-month follow-up period.

The mean absolute LDL-C change from baseline to month 6 was −82.9 mg/dL (95% CI, −96.7 to −69.2), corresponding to a mean percentage change of −46.7% (95% CI, −53.0 to −40.4) (Figure 1).

No treatment-limiting adverse events were reported. Hepatic, renal, creatine kinase, and viro-immunological parameters remained stable during follow-up.

To compare treatment responses between groups, a mixed-design repeated measures ANOVA was performed as an exploratory analysis. A significant group × time interaction was observed for total cholesterol (F = 5.58, *p* = 0.005, partial η^2^ = 0.071), LDL-C (F = 7.28, *p* = 0.001, partial η^2^ = 0.091), and non-HDL cholesterol (F = 6.65, *p* = 0.002), indicating different temporal patterns of lipid reduction between PLWH and HIV-negative controls. Specifically, controls achieved most of the lipid reduction within the first three months and subsequently reached a plateau, whereas PLWH demonstrated a more progressive decline between months 3 and 6. Because the cohorts were not individually matched and the analysis was not adjusted for potential confounders, these findings should be considered exploratory and hypothesis-generating rather than evidence of an HIV-specific pharmacodynamic effect.

The latest available full D:A:D model (2016 update) was used to calculate both 5-year and 10-year cardiovascular risk [18]. The mean 5-year D:A:D score decreased from 10.75 ± 11.75% at T0 to 7.72 ± 7.89% at T3 and 6.76 ± 8.09% at T6. The mean 10-year D:A:D score decreased from 19.53 ± 18.70% at T0 to 14.76 ± 13.93% at T3 and 12.84 ± 14.18% at T6. In a complete-case within-person analysis, the overall change across T0, T3, and T6 was statistically significant for both the 5-year and 10-year D:A:D scores (Friedman test, *p* = 6.03 × 10^−7^ and *p* = 3.11 × 10^−7^, respectively; *n* = 28 and *n* = 29). Pairwise T0-to-T6 reductions were also significant for both models (Wilcoxon signed-rank test, *p* < 0.001 for both). Control D:A:D scores were not calculated because the D:A:D model is HIV-specific. Complete longitudinal cardiovascular risk-score data for the HIV-negative control cohort were not available in the definitive database; consequently, claims regarding between-group risk-score trajectories were not made.

The database-derived correlation between change in LDL-C and change in the 10-year D:A:D score was weak and not statistically significant (Spearman’s rho = 0.245, *p* = 0.177; *n* = 32). The corresponding correlation with the 5-year D:A:D score was also not statistically significant (Spearman’s rho = 0.255, *p* = 0.159). Because total cholesterol and HDL-C contribute to the D:A:D calculation, these associations may partly reflect shared lipid-related information and should not be interpreted as independent causal effects. The corresponding approximate 95% confidence intervals were −0.114 to 0.547 for the 10-year score and −0.103 to 0.554 for the 5-year score (Table 2).

## 4. Discussion

This study shows that low-dose fixed-dose rosuvastatin/ezetimibe substantially improved atherogenic lipid parameters in virologically stable PLWH [11,17,19]. LDL-C decreased by 46.7% at 6 months, and 53.1% of PLWH achieved the target appropriate to their risk category. All patients initiated the combination de novo, and the treatment decision was based on elevated LDL-C and the expected need for substantial LDL-C lowering [15,16,20,21,22].

These findings are broadly consistent with previous work in HIV-positive patients in which ezetimibe added to rosuvastatin produced additional LDL-C lowering compared with rosuvastatin titration, and with the REPRIEVE evidence supporting statin-based prevention in PLWH. However, differences in baseline risk, study design, follow-up, and treatment selection limit direct cross-study comparisons; the present observational data should therefore be interpreted as supportive rather than confirmatory [11,17,19].

The HIV-negative cohort was selected as a clinically relevant comparator with a similar treatment indication and broadly comparable overall cardiovascular-risk burden, rather than as a demographically identical matched population. HIV itself is a cardiovascular risk modifier through persistent inflammation, immune activation, antiretroviral exposure, and metabolic abnormalities [5,16,23]. At the same time, the age, sex, BMI, and comorbidity differences between cohorts may influence lipid response, and the between-group analysis remains exploratory. Residual confounding cannot be excluded.

Both cohorts showed marked reductions in atherogenic lipid parameters, but the temporal pattern differed: controls achieved most of the reduction during the first three months, whereas PLWH showed a more gradual decline through month six. This pattern may be influenced by demographic and metabolic differences, antiretroviral-related factors, treatment timing, or other unmeasured determinants. It should therefore be regarded as hypothesis-generating rather than as evidence of an HIV-specific pharmacodynamic effect.

No participant received a ritonavir- or cobicistat-containing antiretroviral regimen. Ritonavir and cobicistat are pharmacokinetic boosters known to interact with statins and may affect statin exposure. Nevertheless, no treatment-limiting adverse events were observed during follow-up, and hepatic, renal, creatine kinase, and viro-immunological parameters remained stable. The available ART distribution has therefore been reported, while formal regimen-specific analyses were not performed because of limited statistical power [6,24].

Diabetes, hypertension, and other cardiometabolic conditions may contribute to residual cardiovascular risk and should be addressed alongside lipid lowering. The present findings therefore support comprehensive risk-factor management but do not allow attribution of the observed response to a single biological mechanism [6,7,8,21]. The fixed-dose single-pill strategy may also simplify treatment and reduce pill burden; however, adherence was not formally assessed in this retrospective study, so any adherence-related benefit remains hypothetical [25].

The latest available full D:A:D model (2016 update) was used to describe HIV-specific estimated cardiovascular risk. The D:A:D scores themselves decreased significantly over time in PLWH, with a significant overall longitudinal change for both the 5-year and 10-year models (Friedman test, *p* < 0.001 for both). The relationship between LDL-C change and D:A:D change was examined as an exploratory association, particularly because total cholesterol and HDL-C are included in the D:A:D equation and risk scores are surrogate estimates rather than clinical cardiovascular endpoints.

This was a retrospective, single-center study with a small sample size and 6-month follow-up. The control cohort was not individually matched for age or sex, although it had a comparable treatment indication and baseline Framingham risk and all controls were naïve to lipid-lowering therapy. Residual confounding remains possible. The study did not include a statin-monotherapy comparator and cannot establish superiority of rosuvastatin/ezetimibe over sequential treatment. Cardiovascular risk was assessed using surrogate scores rather than clinical events. Larger prospective multicenter studies with appropriately matched or adjusted controls are required. The limited sample size also precluded a reliable multivariable adjustment for the full set of clinically relevant covariates; consequently, the comparative analyses were deliberately retained as unadjusted and exploratory [23].

## 5. Conclusions

Low-dose rosuvastatin combined with ezetimibe was associated with substantial improvement in the atherogenic lipid profile of virologically stable PLWH, including a 46.7% mean LDL-C reduction at 6 months and LDL-C target attainment in 53.1% of participants. Both the 5-year and 10-year D:A:D scores also declined significantly during follow-up, with an overall longitudinal *p* < 0.001 for both models. The different temporal lipid patterns observed in PLWH and HIV-negative controls remain exploratory because the cohorts were not individually matched and complete control risk-score data were unavailable. These findings support the hypothesis that fixed-dose rosuvastatin/ezetimibe may be a useful strategy for selected PLWH requiring substantial LDL-C lowering. Prospective multicenter studies should confirm these findings, compare treatment strategies directly, and investigate whether HIV-related inflammation, antiretroviral exposure, and cardiometabolic factors contribute to differences in lipid kinetics and translate into clinically meaningful cardiovascular benefit.

## Figures and Tables

**Table 1:** Baseline characteristics of PLWH and HIV-negative controls.

Variable	PLWH (*n* = 32)	Controls (*n* = 43)
Age, years	54.0 ± 7.4	63.3 ± 9.3
Male sex, *n* (%)	23 (71.9)	16 (37.2)
BMI, kg/m^2^	25.9 ± 5.3	28.6 ± 5.1
Current smokers, *n* (%)	10 (31.3)	5 (11.6)
Diabetes mellitus, *n* (%)	3 (9.4)	3 (7.0)
Hypertension, *n* (%)	15 (46.9)	28 (65.1)
Antihypertensive therapy, *n* (%)	11 (34.4)	26 (60.5)
Framingham 10-year risk, %	19.6 ± 18.7	20.4 ± 14.3

**Table 2:** D:A:D cardiovascular risk scores during follow-up in PLWH.

D:A:D Score	T0 Mean ± SD	T3 Mean ± SD	T6 Mean ± SD	*p*-Value (Overall Time Effect; Friedman Test)
5-year risk (%)	10.75 ± 11.75	7.72 ± 7.89	6.76 ± 8.09	6.03 × 10^−7^
10-year risk (%)	19.53 ± 18.70	14.76 ± 13.93	12.84 ± 14.18	3.11 × 10^−7^

*p*-values were calculated using complete-case Friedman tests; *n* = 28 for the 5-year model and *n* = 29 for the 10-year model.

## Data Availability

The datasets used and evaluated in this study are available from the corresponding author upon reasonable request.
